# Tumours and Tubercle in Monkeys

**Published:** 1907-06

**Authors:** W. Roger Williams


### TUMOURS AND TUBERCLE IN MONKEYS.

BY

W. ROGER WILLIAMS, F.R.C.S.

---

GREAT interest attaches to the maladies of monkeys, because it is among the highest members of this order that we find the nearest approach in organisation to mankind.

Notwithstanding the immense number of these creatures constantly under observation in the zoological collections of Europe, it is a curious fact that only about half a dozen examples of tumours have hitherto been reported; and their comparative immunity from this kind of malady seems to be a reality.

Thus, some time ago Bland-Sutton[1] examined the bodies of 110 of these animals who had died in the London zoo, but not a single example of any tumour did he find. Subsequently, H. J. Campbell[2] made 38 similar *post-mortem* inspections with the like negative result.

**Cancer.**—Leblanc,[3] however, long ago reported that he had met with instances of malignant tumours in monkeys, and I expect that they do occasionally occur, but I can cite only two modern instances.

The first of these is due to Goodhart,[4] who found "cancer" of the pituitary body in an Anubis baboon from the London zoo, where the animal had long been a familiar denizen. The tumour—a large, ragged-looking object—occupied the pituitary fossa, which it had eroded, and some of the adjacent structures were infiltrated. Histologically, it comprised "large epithelial-like cells arranged in some sort of an alveolar manner." There were no secondary deposits. This tumour, together with the brain and skull, are preserved in the museum of the Royal College of Surgeons.

1 *Lancet*, 1883, ii. 276. 2 *Guy's Hosp. Rep.*, 1891, xlviii. 19.

3 *Clin. Vet.*, 1843, Aug., p. 343.

4 *Tr. Path. Soc. Lond.*, 1883, xxxvi. 36.

The second instance was met with in a bonnet monkey, only eight months old, by Bland-Sutton,[1] the tumour being an intra-ocular glioma, consisting chiefly of small round cells.

In this connection reference may be made to the attempts of Metchnikoff, Shattock and Ballance and others, to transmit human cancer experimentally to monkeys, all of which experiments failed.

Thus monkeys, like human savages, seem to have very little proclivity to cancer.

Here it may be remarked that the alimentary propensities of these animals are predominantly frugivorous; but a good many of them are not averse to animal food when they can get it; some kinds are insectivorous, and others feed upon almost anything they can get. Like mankind, many species have a singular liking for birds and their eggs, as alimentary dainties.

**Non-malignant Tumours.**—With regard to non-malignant tumours, the available data are exceptionally meagre. Bland-Sutton has met with an instance of leio-myomatous thickening in the uterus of a baboon, which had some resemblance to myoma; and the same observer has also seen a fatty, tumour-like mass in the vicinity of each testis of a monkey, with hermaphroditic malformation.

According to Otto, exostosis is not uncommon at the tip of the tail of long-tailed monkeys; and in the museum of the Royal College of Surgeons of Ireland the hand of a monkey is preserved, showing a spongy exostosis of the first phalanx of the little finger.

Monkeys are also subject to hydatid cysts.

These few examples practically exhaust our present knowledge of non-malignant tumours in monkeys.

**Tubercle.**—It is an ancient belief, that monkeys kept in captivity are very prone to tubercle, and some years ago a mild sensation was experienced when Bland-Sutton[2] flatly contradicted this cherished conception. In justification of his contention, he appealed to the records of 110 *post-mortem* inspections of monkeys which had died in the London zoo, and comprised only three instances of tubercle.

[1] *J. Anat. & Physiol.*, 1885, xix. 449. [2] *Loc. cit.*

He found, however, that these animals had experienced very heavy mortality from diseases of the lungs, the list comprising 22 examples of bronchitis, 11 of pneumonia, &c.

Some years later, H. J. Campbell[1], as the result of similar work in the same field, arrived at exactly the opposite conclusion, having found that tuberculous disease was very frequent in these monkeys. Thus, no less than 20 of the 38 bodies he examined presented well-marked tuberculous lesions. In addition to these, there were also many cases of broncho-pneumonia.

It is evident that these discrepancies depend largely upon diversity as to the criterion of tubercle. Viewing the matter in this light, we shall probably be right in maintaining the validity of the old belief.

In support of this, reference may be made to the observations of Dr. A. J. Harrison, who has long been connected with the management of the fine collection of animals in the Clifton zoo. He says[2]:—"Monkeys are very liable to chest affections, and there can be no question that we have lost a great many from tuberculous disease of the lungs. They seem very prone to pleurisy, and adhesions are frequently found with and without tuberculous masses in the lungs, but actual cavities do not seem to be frequent. Monkeys seem to be particularly prone to tubercle."

It accords with the foregoing, that Lydia Rabinowitsch[3] has lately found many instances of tubercle among the monkeys that died in the Berlin zoo; and of 36 cases in which these lesions were specially examined *ad hoc*, in nearly three-fourths the type of tubercle was human; examples of bovine, avian and mixed types were only occasionally met with.

It has likewise been proved, that monkeys are very susceptible to the experimental inoculation of both the human and bovine forms of tubercle, as the experiences of Dieulafoy, Krishaber, Dungern and others testify.

Of like import is the common occurrence of specimens of simian tuberculous disease in museums, such as that of the Royal

[1] *Loc. cit.* [2] *Bristol M.-Chir. J.*, 1894, xii. 285.

[3] *Deutsche med. Wchnschr.*, 1906, xxxii. 866.

College of Surgeons of Ireland, which have good collections illustrative of the pathology of these animals.

According to Woods Hutchinson,[1] monkeys in their native forests are but little prone to tubercle; but in captivity it is difficult to procure specimens free from the disease. Thus of 45 monkeys that died in captivity at the London zoo (1898 to 1899) 17 died of tubercle, or 38 per cent. Food habits have much to do with tubercle mortality; for of Hutchinson's animals 35 were vegetarian Catarrhines, and it was among these that all the 17 deaths occurred; whereas, not one of the 10 deaths among the Platyrrhine monkeys, who had taken a fair amount of animal food, was due to tubercle.

